# Provider fidelity in tuberculosis screening practices among adolescents and adults living with HIV in public health facilities in Tanzania: a cross-sectional evaluation

**DOI:** 10.3389/fpubh.2025.1688829

**Published:** 2025-11-19

**Authors:** Lucas L. Shilugu, Lawrencia D. Mushi, Mackfallen G. Anasel

**Affiliations:** Department of Health Systems Management, School of Public Administration and Management, Mzumbe University, Morogoro, Tanzania

**Keywords:** tuberculosis, TBHIV, screening, algorithm, fidelity, evaluation

## Abstract

Tuberculosis (TB) remains the leading cause of morbidity and mortality among people living with HIV (PLHIV) in high-burden countries like Tanzania. Despite national and global guidelines recommending routine TB screening at every clinical encounter, missed and delayed TB case notifications persist, suggesting gaps in screening practices. This study evaluated the implementation fidelity of the TB screening algorithm and associated factors within routine HIV care in public health facilities in Geita, Tanzania. A facility-based cross-sectional study was conducted, involving the extraction of data from 423 client treatment records and observation of 423 screening sessions. Simple random and systematic sampling methods were employed to select the records and sessions, respectively. Descriptive and inferential analyses were performed using Excel and Stata. Modified Poisson regression with robust variance was used to estimate prevalence ratios (PRs) to determine factors associated with two binary outcomes: (1) consistent TB screening over 12-month period, and (2) correct utilization of the screening tool. TB screening was documented in 82.8% of clinical encounters. Overall, 70.7% of clients were screened at every encounter, and 75.4% screened at their most recent visit. Laboratory investigations were recorded in 94% of presumptive cases, with all confirmed TB cases initiated on treatment. Additionally, 80.6% of eligible clients were initiated on TB preventive therapy (TPT). The WHO Four-Symptom Screening (W4SS) was widely used (98.8%), and the tool was correctly administered in 62% of the sessions observed. Factors associated with inconsistent screening included age 40–49 years [Adjusted Prevalence Ratio (aPR) = 0.82; *p* = 0.046], age ≥50 years (aPR = 0.76; *p* = 0.025), suppressed viral load (aPR = 0.63; *p* < 0.001), monthly drug refill model (aPR = 0.55; *p* = 0.006), refill by treatment supporter (aPR = 0.09; *p* < 0.001), being traced back from a lost to follow up (aPR = 1.38; *p* = 0.019), and absence of prior TB (aPR = 0.81; *p* = 0.001). The correct use of the W4SS tool was less likely at PMTCT clinics (aPR = 0.55; *p* < 0.001). Although TB screening is widely integrated into HIV care, fidelity to the screening algorithm remains suboptimal in the initial stages of symptom screening. The completion of the algorithm cascade was well-implemented. Fidelity at earlier stages of the algorithm, such as the consistent and correct use of the W4SS, should be enhanced by strengthening provider capacity and routine monitoring to improve adherence to the protocol.

## Introduction

Tuberculosis (TB) and Human Immunodeficiency Virus (HIV) co-infection continues to be a significant global public health problem, especially in Sub-Saharan Africa (SSA), where these infections are prevalent (1–5). The co-infection persists as people living with HIV (PLHIV) are more likely to suffer from TB, where the lifetime risk of developing active TB among PLHIV may be 20–30 times higher than in people without HIV (6–9), and about 30% of PLHIV do have latent TB active (6, 10). This happens as HIV lowers the immunity against TB, leading to increased active TB infection, re-infection, or reactivation, and increases the risk of TB progression from latent TB to active TB disease (8).

Despite the increased access to antiretroviral therapy (ART), mortality in PLHIV is still high, and TB remains the leading cause of death, accounting for one-third of global deaths in this population (5, 6, 11–14). Globally, an estimated 167,000 PLHIV died from TB in 2022, a number which has been declining steadily (15). Previous studies in SSA have observed that nearly 80% of HIV-related mortality cases are associated with TB (6), and the likelihood of mortality increases among PLHIV with this co-infection (7, 16).

Tanzania is among the 30 countries classified by WHO as high TB/HIV burden countries (2). Thirty per cent of notified TB cases in 2018 were co-infected with HIV, and an estimated 33,000 people who developed TB in 2019 were co-infected with HIV (17, 18). Moreover, as in other countries where TB and HIV are prevalent, TB is the leading cause of death in this population. In a longitudinal study in Tanzania, PLHIV with TB co-infection had 40% higher mortality than those without TB (8). In 2021, the estimated number of HIV-infected people who died from TB in Tanzania was 7,800 (19).

Nevertheless, TB is preventable and curable if treatment is received quickly and appropriately (20). Thus, rapid and accurate diagnosis and the use of effective anti-TB treatments are priority tools not only for minimizing morbidity and mortality but also for mitigating the spread of TB among the population (16, 20). With the treatments currently recommended by the WHO, about 85% of people with TB can be cured if they are diagnosed and initiated on treatment in a timely manner (1, 15). Thus, “People living with HIV should be systematically screened for TB disease at each visit to a health facility” (14). Systematic TB screening among PLHIV aims to detect TB disease early to minimize avoidable delays in diagnosis and initiation of treatment, thereby reducing the risk of unfavorable treatment outcomes, health sequelae, adverse social and economic consequences, and reducing transmission of the disease (14, 21).

The WHO recommends the use of the parallel screening algorithm which requires all adolescents and adults living with HIV to be screened for symptoms and signs of TB using the WHO four symptom screening (W4SS) and/or Chest X-ray (CXR) on every clinical encounter. Individuals who have a positive answer to any of the four W4SS questions (cough of any duration, night sweats, fever or weight loss) or suggestive CXR should undergo further TB diagnosis according to the guidelines (18, 21), those who are diagnosed with TB should start treatment immediately (18, 21). After ruling out TB, the person living with HIV has to be provided with TB preventive treatment (TPT) as recommended (14, 18, 21).

Despite incorporation into the national guidelines and widespread use of the algorithm, there remains an unacceptable number of missing TB cases, and the infection continues to be the leading cause of death among PLHIV in Tanzania (5, 8, 13, 22, 23). TB decreases by only 5% on average each year in Tanzania; this slow decline, marked by substantial gaps, persists in the timely detection and treatment of TB cases. As a result, achieving reduced TB transmission and meeting the END TB targets may not be feasible in the near future (1, 15, 23). In 2021, it was estimated that 24,000 incident TB patients were HIV positive; however, only 64% were diagnosed, revealing significant gaps in TB diagnosis among PLHIV (24).

Additionally, Tanzania has remained one of the 30 high TB/HIV burden countries for nearly a decade now, since it was listed in 2016 (2). This indicates ongoing challenges in current mechanisms to diagnose and treat TB in PLHIV and that the TB screening intervention among PLHIV is hardly achieving the desired early detection and prevention of TB in this population. An analysis of 2012–16 routine DHIS2 data showed undesirable TB screening outcomes among PLHIV in the country (12). Thus, improvements and better mechanisms need to be continuously thought out to ensure early TB diagnosis and treatment toward the “*End TB Strategy 2023–2030*” and the “*Global Plan to End TB by 2030*” (15, 25). To do this, a careful investigation is needed to understand the fidelity of implementing the parallel screening algorithm at health facilities. Fidelity to the screening process refers to the extent to which TB screening is delivered in adherence to the screening algorithm as it was originally designed and recommended by the WHO, and it is important to determine whether the intervention works as intended (26, 27). Therefore, this evaluation assessed the implementation process of the screening algorithm for detecting TB among adolescents aged 10 to19 years and adults living with HIV in public health facilities in Geita region, Tanzania.

## Materials and methods

### Evaluation design and setting

This process evaluation used a cross-sectional study design with a quantitative approach, which was suitable for descriptive and analytical analysis of the level of fidelity to the screening algorithm and associated determinants (28). A facility-based cross-sectional study design has also been employed in other process evaluations that investigated a similar topic (29, 30). The study was carried out in public health facilities in Geita region, Tanzania. Geita is among the regions with higher burden of TB in the country and has a higher (4.9%) HIV prevalence, exceeding the national average (4.4%), with a lower (72.2%) HIV viral load suppression rate, which is below the national average (78.1%) (31).

### Participants and selection procedures

The study involved adolescents aged 10–19 years and adults living with HIV who were receiving antiretroviral therapy in public health facilities within Geita Region. The required sample size was determined using a single population proportion formula, as applied in similar studies (29, 30, 32). The expected proportion of compliance with the use of the algorithm in TB screening was set at 50% as there was no prior data. The confidence level was set at 95% (1.96), and the margin of error was set at 5% as used in previous studies that investigated a related topic (29, 32). Plugging in the formula, this yielded a sample of 384. A non-response rate of 10% (33) was added to make the final sample size 423.

The required sample was obtained through a multi-stage stratified sampling method. Initially, simple random sampling was used to select three district councils from the six councils in the region. Next, a list of all health facilities in the three selected councils was obtained and entered into Excel for Microsoft 365, and then restricted simple random sampling was performed using the computer software (Excel) to select eight health facilities for the study; the restrictions were made to ensure that two district hospitals, three health centers, and three dispensaries were chosen from the list.

Then, the total sample size (423) was distributed among the sampled health facilities in proportion to the number of clients on ART at each facility. This was achieved using a probability proportional to size (PPS) sampling strategy, which was carried out after determining the number of clients in the relevant age group from the CTC2 database for the selected facilities. PPS was also employed to allocate the sample size across units such as the care and treatment center (CTC) and Prevention of Mother-to-Child Transmission (PMTCT). This approach was used to guarantee equal representation of members within the sampling frame.

After determining the sample size for the CTC and PMTCT departments in the eight selected health facilities, simple random sampling was employed to select the client treatment records (CTC2 files) for review. Systematic sampling was used to choose the observed screening sessions based on clients who were present at the clinic on the day.

### Variables and measurements

The outcome variable – fidelity to the screening algorithm – was measured in three parameters: (1) consistent screening, defined as whether clients were screened at every clinical encounter within 12 months, recorded as “yes” if the client was screened during all visits and “no” if not consistently screened; and (2) whether the recommended steps in the TB screening algorithm were completed, recorded as “yes” if presumptive TB cases had laboratory results, diagnosed TB cases were started on treatment, and eligible individuals were commenced on TB preventive therapy, and “no” otherwise. Data on these parameters were collected through document review of client CTC2 files. The third outcome variable was assessed through direct observation during screening sessions: (3) correct administration of the W4SS tool, recorded as “yes” if all four recommended questions were asked correctly, and “no” if any were omitted or asked incorrectly. Probing was not considered; our observation focused solely on whether the questions were asked correctly or not.

Independent variables obtained from treatment records included age (in years), sex (male/female), occupation (formal employment, peasant, mining, student, or other), marital status (single, married, cohabiting, divorced, separated, or child under 15 years), type of health facility (hospital, health center, or dispensary), clinic type (CTC or PMTCT), WHO HIV clinical stage, client category (unstable or stable [has no opportunistic infections and has suppressed viral load]), duration on ART (within 6 months or 7 months and above), drug collection model (monthly, three-month, or six-month facility-based or community-based), history of TB (previous TB or never had TB), TPT status (completed, interrupted, currently on TPT, or not started), and visit type (scheduled, unscheduled, visit by treatment supporter, or traced after loss to follow-up). Independent variables observed during screening sessions included sex, type of health facility, and clinic type.

### Data collection

Data were extracted from 423 client treatment records (CTC2 files) of March 2024 – February 2025. Concurrently, 423 screening sessions were covertly observed to assess the extent to which health care providers correctly use the W4SS screening test. Document review and observation had been used as data collection methods in similar facility-based process evaluation (29). Data were collected using an abstraction checklist and observation checklist designed in Kobo Toolbox, and data collection was carried out using the Kobo Collect Android application installed on smartphones. A team of two research assistants was recruited and adequately oriented on the study objectives, data collection procedures, and ethical considerations.

The tool was piloted with 30 participants across two non-study health facilities to assess its capacity to gather the information needed for the evaluation objectives and to familiarize data collectors with its use. The questions were revised following the pilot study, including adding the “not recorded” response for missing records in reviewed documents, incorporating additional skip logic in the Kobo Toolbox system for questions requiring responses to skip to specific questions, and adding a question about the presence of the screening form in the client file, which was previously omitted. For the observation component, the tool was refined by removing some questions that were difficult to answer through covert observation, such as client occupation, marital status, education level, and other clinical information like duration on treatment, model of drug collection, viral load suppression status, history of TB, and TPT status. The data collection process was also adjusted to ensure data acquisition, which involved rescheduling collection days to coincide with clinic days for CTC and PMTCT. Data were collected in March 2025.

### Data management

All finalized forms were cross-checked for completeness while at the health facility. Completed questionnaires were sent and securely saved on the password-protected Kobo Toolbox. The final data file was exported in a Microsoft Excel format and saved on a password-protected computer accessible only to the principal investigator. A copy of the data set was saved in STATA (dta) format for cleaning and analysis.

During data cleaning, all string variables were converted into a format that STATA can read (numerical values). String variables were transformed by creating new numerical variables; the original values were replaced, recorded, labeled appropriately, and cross-checked with the original variables using relevant STATA version 17 commands. For the regression analysis, age was categorized into adolescents (10–19 years) and adults (20–29 years, 30–39 years, 40–49 years, and 50 and above). The adult age groupings were categorized at interval of 10 years for uniformity to adolescence age interval. The adolescent–adult dichotomy aligned to the programmatic importance, as TB screening and HIV service delivery strategies often differentiate between these two populations in terms of clinical management, communication approaches, and adherence support needs (34). The age groups were established by creating a new variable, “Age_Category,” which took values from the variable age, and then the categories were assigned correctly using the STATA *recode* command. For the regression analysis, all data were coded into dichotomous categories, with dummy variables created for all variables that had more than two responses. Responses with missing values, including those with missing records from the treatment (CTC2) files, were marked as missing and subsequently excluded in the analysis within STATA 17. A do-file was saved and shared alongside the dataset for cross-checking by another independent evaluator experienced in quantitative data management and analysis.

### Data analysis

Both descriptive and inferential analyses were performed using Microsoft Excel (Office 365) and Stata version 17. Microsoft Excel was used to generate visual presentations, such as pie charts, included in the results. Further descriptive statistics were performed in Stata to analyze key demographic characteristics of participants and to compute the proportions and levels of fidelity to the TB screening algorithm. In the descriptive analysis, the continuous variable age was summarized using the median and interquartile range (IQR), as the data were not normally distributed (35, 36). Although the distribution curve appeared approximately bell-shaped, the Shapiro–Wilk test for this variable indicated non-normality (*p* = 0.001), justifying the use of non-parametric summaries (37). All other variables, including categorized age groups, were presented as proportions, as the data were categorical. The difference in proportion distributions between adolescents and adults, as well as between males and females was assessed using the Chi-square (*χ*^2^) test, as variables were categorical. To ensure the appropriateness of the Chi-Square test, expected cell frequencies were examined. If any expected cell count was less than 5 or if a cell had zero observation, Fisher’s Exact Test was used instead.

Regression analyses assessed two distinct outcomes: (1) consistency of TB screening over a 12-month period (periodic prevalence measured as “yes” consistently screened or “no” for otherwise), and (2) correct utilization of the W4SS screening tool (a cross-sectional point-prevalence measured as “yes” the W4SS questions correctly asked or “no” if any were omitted or incorrectly asked). These two outcomes were selected to provide a comprehensive understanding of fidelity in the screening practices by capturing both adherence to consistent use of the screening tool over time and accuracy in its application during clinical encounters. We estimated prevalence ratios (PRs) using modified Poisson regression with robust variance, a method recommended for cross-sectional studies with common outcomes (33, 38–40). Initially, log-binomial regression was utilized for bivariable analysis. However, due to convergence issues in the multivariable model, Modified Poisson regression with robust variance was adopted as an alternative, providing consistent and interpretable estimates of prevalence ratios (33, 41). For methodological consistency, we applied Modified Poisson regression to all bivariable analyses.

Both bivariable and multivariable Poisson regression analyses were conducted. All 14 predictors were tested in the bivariable analyses. In the multivariable analyses, variables considered clinically important for TB screening among PLHIV, as well as those with a *p*-value ≤0.2 in the Chi square test, were considered in the model. Thirteen variables were initially included in the model, with one variable, ‘occupation’, excluded after showing high correlation with the variable ‘sex’ in the Chi-square test for multicollinearity (*p* = 0.001). A stepwise backward elimination method was employed, as used in other studies (42), where non-significant predictors were removed one-by-one while monitoring the Pseudo R^2^ until only five statistically significant variables remained. Nonetheless, three clinically important variables – TP preventive treatment status, client stability status, and sex – were retained in the model due to their clinical relevance to TB disease. The relationship between predictor variables and the binary outcomes (consistent TB screening and correct application of the W4SS questions) was assessed using a generalized linear model with a Poisson distribution, log link function, and robust (sandwich) standard errors, as recommended for binary outcomes with common events (33, 40).

The model is expressed as:


$$\log(E[Y|X])=\beta_{0}+\beta_{1}X_{1}+\beta_{2}X_{2}+\cdots +\beta_{k}X_{k},$$

Whereas *E*[*Y/X*] denotes the expected probability of consistent screening or correct asking of the W4SS questions. The exponential of the estimated coefficients ( $e^{\beta_{i}}$ ) gives the Prevalence Ratio for each predictor. Y is the binary outcome, defined as: (1 = Consistent screening; 0 = Otherwise); and (1 = Correct asking the W4SS questions; 0 = Otherwise). *β_0_* = Is the intercept, *β*_*1*,_ *β*_*2*,_ *β_k_* = Poisson regression coefficient of *X*_*1*,_ *X*_*2*,_ *X_k_*, respectively., *X*_*1*,_ *X*_*2*,_ *X_k_* = Independent variables.

The assumptions of Poisson regression were evaluated, including testing for multicollinearity among independent variables, model’s goodness of fit and explanatory power. First, we assessed multicollinearity among independent variables using the chi-square test for categorical variables, and excluded one variable (‘occupation’) that showed a significant correlation with ‘sex’ to enhance model stability. Sex was retained over occupation because it is a more fundamental biological determinant relevant to TB screening practices. Second, we examined the model fit using the Pearson goodness-of-fit chi-square test. The model yielded a chi-square statistic of 117.67 with 395 degrees of freedom (*p* = 1.000), indicating no evidence of lack of fit or overdispersion. The ratio of the Pearson chi-square statistic to its degrees of freedom (0.30) further suggested adequate model fit. In addition, the use of robust variance estimators accounted for any minor deviations from the equidispersion assumption, ensuring valid standard errors and confidence intervals. For the primary outcome – consistent TB screening over 12-month period – the final multivariable Modified Poisson regression model included 413 observations (excluding 10 with missing values), it had a Pseudo R^2^ of 59.23% and was statistically significant (*p* < 0.001), indicating that the included predictors explained more than half of the variability in consistent TB screening practices among participants. For the second outcome – correct use of the W4SS – the final model included 418 observations (excluding 5 with missing values), it had a Pseudo R^2^ of 5.8% and was statistically significant (*p* < 0.001). Statistical significance was determined at a 95% confidence interval expressed as a *p* value less than 0.05. Data were presented in tables and figures (pie chart).

### Validity and reliability

#### Validity

To improve the face and content validity of the instruments, the questionnaire was carefully designed and reviewed by an independent expert. Pilot testing was conducted to evaluate the tool’s effectiveness, allowing for adjustments and refinements to further improve validity. The questions in the questionnaire were developed based on measurements used in previous studies (12, 43). By drawing on these established frameworks and validated measures, we ensured that the questionnaire covered key aspects to meet the research objectives. These steps collectively helped strengthen the face, content, and construct validity of the research instruments.

#### Reliability

Reliability was ensured through the development of a detailed research protocol, which was presented and approved by the research board of Mzumbe University. The approved protocol was strictly followed throughout the research process to maintain consistency in data collection procedures. The methodology was well elaborated, and the analysis process was rigorously conducted using Stata to ensure consistency, replicability, and generalisability of the findings. Research assistants were trained and supervised to guarantee uniformity in administering the tools and following data collection procedures. The focus on methodological consistency and the use of established tools and standard measurements ensured that the study adhered to best research practices, thereby enhancing the reliability of the findings.

## Results

### Characteristics of participants

A total of 423 treatment records were reviewed. The median age was 33 years (IQR = 14), with the youngest being 11 and the oldest 65 years. A higher proportion of clients in the records were aged 30–39 years (34.7%) and 20–29 years (26.6%). Males were older than females; for example, 15.8% of males were aged 50 years and above compared to 5.6% of females (*p* = 0.002). Nearly three quarters (70.4%) were married. There was no significant difference in marital status proportions across participant sex (*p* = 0.806). More than three quarters (78.5%) of clients were peasants. More females than males were peasants (86.4% vs. 69.4%), while more males than females worked in artisanal mining (18% vs. 2.8%; *p* < 0.001). Over half (57.3%) of the clients received care at health centers; fewer males than females attended health centers (52.1% vs. 61.5%), while more males than females attended hospitals (33.2% vs. 23.3%; *p* = 0.074). The majority (82.5%) attended CTCs. All males and a majority (68%) of females attended CTCs (*p* < 0.001) (Table 1).

**Table 1:** Demographic characteristics of 423 participants whose treatment records were reviewed in public health facilities engaged in the study in Geita, March 2025.

Variable	Categories	Total		Male		Female		*p*
		*N*	%	*N*	%	*N*	%	
Age (in years)	10–19	36	8.5	17	8.9	19	8.2	0.002
	20–29	113	26.6	44	23.1	69	29.5	
	30–39	147	34.7	55	29.0	92	39.4	
	40–49	84	20.0	44	23.2	40	17.3	
	50+	43	10.2	30	15.8	13	5.6	
Marital status	Single	72	19.2	35	20.6	37	18.1	0.806
	Married	264	70.4	117	68.8	147	71.7	
	Divorced/Separated	39	10.4	18	10.6	21	10.2	
	Not recorded	47*	-	18	-	28	-	
Occupation	Peasant	256	78.5	104	69.4	152	86.4	0.001**
	Formal employment	2	0.6	2	1.3	0	0	
	Artisanal mining	32	9.8	27	18.0	5	2.8	
	Petty trade	25	7.7	12	8.0	13	7.4	
	Students	11	3.4	5	3.3	6	3.4	
	Not recorded	96*	-	38	-	57	-	
Type of health facility	Dispensary	63	14.9	28	14.7	35	15.2	0.074
	Heath center	242	57.3	99	52.1	143	61.5	
	Hospital	118	27.8	63	33.2	55	23.3	
Type of clinic	CTC	349	82.5	190	100.0	159	68.0	0.001**
	PMTCT	74	17.5	0	0	74	32.0	

*N* (Number), % (Percentage), *p* (*p* value), *(missing records not included in calculation of percentages), **(Fisher’s exact test – one cell has zero observation).

The highest proportions of clients were in WHO HIV clinical stage two (40.1%) and clinical stage one (39.9%). More male than female clients were at clinical stage two (44.2% vs. 36.8%), while more female than male clients were at clinical stage one (48% vs. 30%; *p* < 0.001). The majority (89.7%) had been on antiretroviral treatment (ART) for more than 6 months, and 80.1% had achieved HIV viral load suppression. The duration on ART and the HIV viral load suppression status showed no significant difference in distribution between male and female clients (*p* > 0.05). More than half (68.2%) of the clients were classified as stable. More male than female clients were stable (77.9% vs. 60.2%; *p* < 0.001). Most (85.3%) of the clients had no history of TB. Fewer male than female clients had no TB history (79.7% vs. 89.8%; *p* = 0.014). Nearly 9 in 10 (87.4%) had completed TB preventive treatment (TPT). The TPT status showed no significant difference in distribution across sex categories. Half (50.9%) of the clients were on a six-month facility-based model of drug collection, while the majority (70%) attended a scheduled visit during their most recent clinical encounter. More male than female clients were on the six-month facility-based model (56.8% vs. 45.9%; *p* < 0.001). The clinic visit type did not differ significantly between male and female clients (Table 2).

**Table 2:** Clinical characteristics of 423 participants whose treatment records were reviewed in public health facilities engaged in the study in Geita, March 2025.

Variable	Categories	Total		Male		Female		*p*
		*N*	%	*N*	%	*N*	%	
HIV illness stage	Stage one	168	39.9	57	30.0	111	48.0	0.001
	Stage two	169	40.1	84	44.2	85	36.8	
	Stage three/four	86	20	49	25.8	37	15.2	
Duration on ART	New on treatment (within 6 months)	44	10.3	18	9.5	26	11.2	0.552
	Continuing treatment (above 6 months)	379	89.7	172	90.5	207	88.8	
HIV Viral load suppression status	Not suppressed	84	19.9	40	21.1	44	19.0	0.608
	Suppressed	339	80.1	150	78.9	189	81.0	
Client stability status	Stable client	288	68.2	148	77.9	140	60.2	0.001
	Unstable client	135	31.8	42	22.1	93	39.8	
History of TB	Never had TB	352	85.3	149	79.7	203	89.8	0.014
	On TB treatment	22	5.3	13	6.9	9	4.0	
	Previous TB	39	9.4	25	13.4	14	6.2	
	Not recorded*	8	-	1	-	7	-	
TB preventive treatment (TPT) status	Completed TPT	360	87.4	166	88.8	194	86.2	0.624
	On TPT	35	8.5	13	6.9	22	9.8	
	Not started TPT	11	2.7	6	3.2	5	2.2	
	Interrupted TPT	6	1.4	2	1.1	4	1.8	
	Not recorded*	9	-	1	-	8	-	
Model of drug collection	Facility monthly base	139	33.0	42	22.1	97	41.9	0.001**
	Facility 3 month	67	15.9	40	21.1	27	11.6	
	Facility 6 month	216	50.9	108	56.8	108	45.9	
	Community 6 month	1	0.2	0	0	1	0.43	
Visit type on last clinical encounter	Scheduled visit	296	70.0	125	65.8	171	73.2	0.159
	Unscheduled visit	86	20.4	44	23.2	42	18.2	
	Traced back after LTF	13	3.0	9	4.7	4	1.7	
	Treatment supporter	28	6.6	12	6.3	16	6.9	

*N* (Number), % (Percentage), *p* (*p* value), *(missing value not included in calculation of percentages), **(Fisher’s exact test – one cell has zero observation, and 2 cells contain less than 5 expected frequencies).

For the observation part, a total of 423 screening sessions were observed. More than half (54.6%) of the clients in the observed TB screening sessions were female. The majority (61.5%) were at the health center. Nearly three-quarters (71.9%) of the clients attended CTCs. All males and the majority of females (84.6%) were at the CTCs (*p* < 0.001) (Table 3).

**Table 3:** Descriptive characteristics of participants for the observed TB screening sessions in public health facilities engaged in the study in Geita, March 2025 (N = 423).

Variable	Categories	Total		Male		Female		*p*
		*N*	%	*N*	%	*N*	%	
Type of health facility	Dispensary	53	12.5	25	13.0	28	12.1	0.287
	Heath center	246	58.2	104	54.2	142	61.5	
	Hospital	124	29.3	64	32.8	61	26.4	
Type of clinic	CTC	358	84.6	192	100.0	166	71.9	0.001**
	PMTCT	65	15.4	0	0	65	28.1	

*p* (*p* value), **(Fisher’s exact test – one cell has zero observation).

### Screening for TB on every clinical encounter

A total of 1,677 clinical encounters were recorded over a 12-month period among individuals whose treatment records were reviewed. TB screening was conducted in 1389 of these encounters, representing 82.8%. About three quarters (70.7%) of the individuals were screened for TB during all their clinical encounters, while three quarters (75.4%) were screened in their most recent encounter. The most commonly used screening test was the W4SS, applied in 98.1% of the cases reviewed. W4SS screening forms were available in the majority (95.3%) of the reviewed CTC2 treatment records. Across all categories, there was no statistically significant difference in TB screening distribution between adolescents and adults (*p* > 0.05) (Supplementary Table 1).

### Utilization of the W4SS screening test for detecting TB

**Figure 1:**
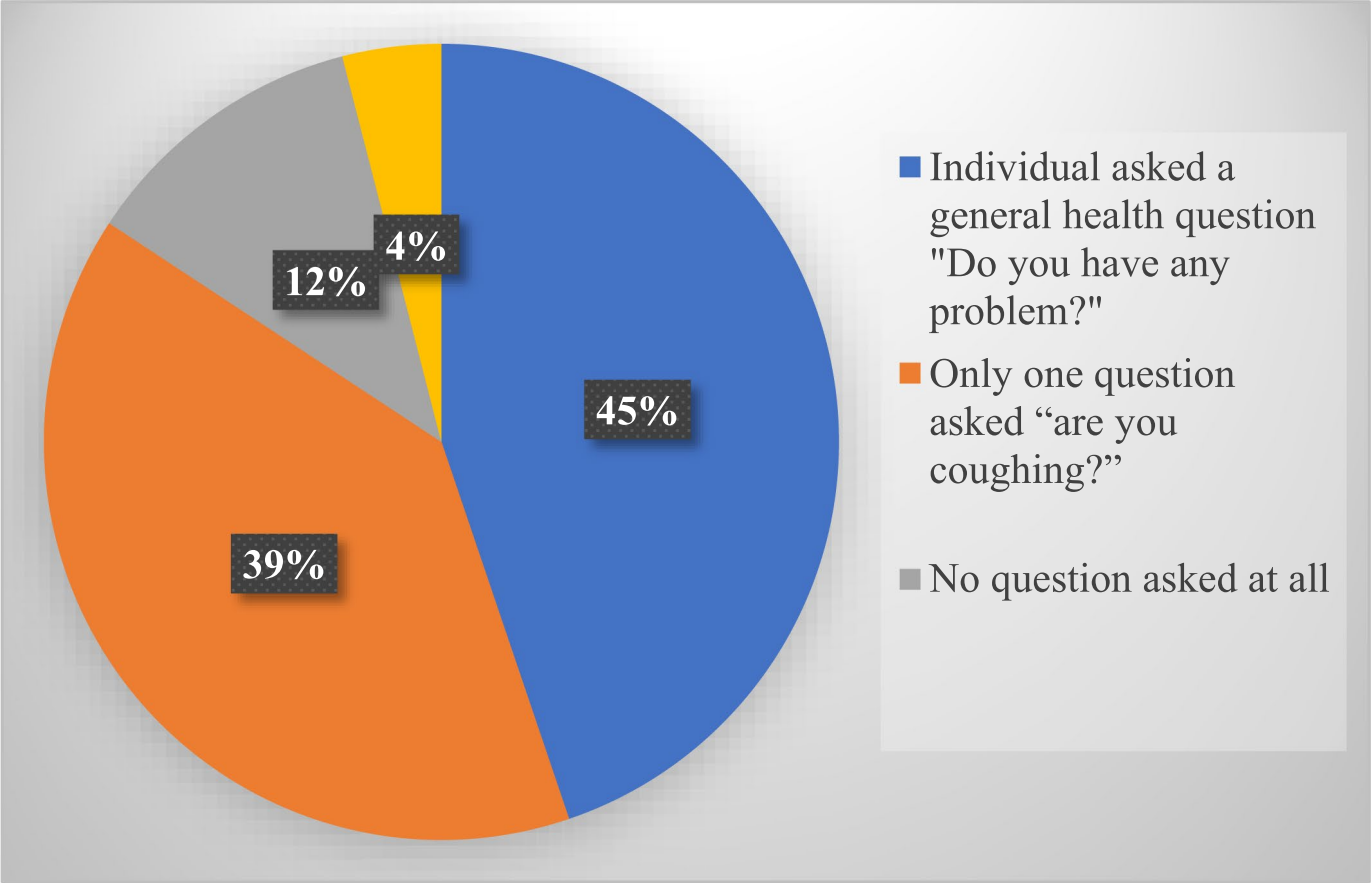
The common non-use of W4SS TB screening practices observed among healthcare providers screening adolescents and adults living with HIV in public health facilities in Geita region, March 2025 (*N* = 159).

The WHO Four-Symptom Screening (W4SS) was widely used in 98.8% of the sessions observed. Healthcare providers correctly asked the W4SS questions in 62% of the screening sessions (Table 4). The common instances of non-use of the W4SS TB screening practices observed among healthcare providers were: asking the client a general health question – ‘Do you have any problem?’ (42%); asking clients only one question about their coughing status – ‘Are you coughing?’ (39%); not asking clients any TB screening question at all (12%); and treatment supporter coming for a refill (4%) (Figure 1).

**Table 4:** The extent to which healthcare providers correctly use the W4SS screening test to detect TB among adolescents and adults living with HIV in public health facilities in Geita region, March 2025 (*N* = 423).

Variable	Categories	Total		Male		Female	
		*N*	%	*N*	%	*N*	%
Screening test used	W4SS	418	98.8	187	97.4	231	100
	CXR	5	1.2	5	2.6	0	0
The healthcare provider asked the four recommended W4SS screening test questions	No	159	38.0	64	32.2	95	41.1
	Yes	259	62.0	123	65.8	136	58.9
	Individual was screened on Chest X-ray*	5	-	5	-	0	-

*N* (Number), % (Percentage), *p* (*p* value), *(not included in calculation of percentages).

### Healthcare providers completion of the recommended steps of the screening algorithm for detecting TB

Laboratory investigation results were recorded in the majority (94%) of the TB presumptive client treatment records reviewed. All (100%) individuals diagnosed with TB were initiated on the recommended TB treatment. Additionally, 80.6% of the clients eligible for TB preventive treatment (TPT) were started on TPT during the most recent clinical encounter. In all categories, there were no statistically significant differences in completion of the algorithm cascade between adolescents and adults (*p* > 0.05) (Supplementary Table 2).

### Factors associated with the implementation fidelity of the TB screening algorithm for detecting TB

Table 5 shows the bivariable and multivariable Modified Poisson regression analyses of factors associated with consistent TB screening over a 12-month period. In the multivariable Poisson regression model, an increase in age was significantly linked to a lower prevalence of consistent TB screening at every clinical encounter. Specifically, adults aged 40–49 years had an 18% lower prevalence of consistent TB screening at each clinical encounter compared to adolescents [Adjusted Prevalence Ratio (aPR) = 0.82; 95% CI = 0.68–0.99; *p* = 0.046], and adults aged 50 years or older had a 24% lower prevalence of consistent screening compared to adolescents (aPR = 0.76; 95% CI = 0.60–0.97; *p* = 0.025). Clients with suppressed HIV viral load had a 37% lower prevalence of being consistently screened for TB at every clinical encounter compared to those with unsuppressed HIV viral load (aPR = 0.63; 95% CI = 0.49–0.79; *p* < 0.001). Individuals without a history of TB had a 19% lower prevalence of consistent TB screening at every clinical encounter compared to those with a history of TB (aPR = 0.81; 95% CI = 0.71–0.92; *p* = 0.001).

**Table 5:** Modified Poisson regression of factors associated with consistence TB screening at every clinical encounter over a 12-month period among adolescents and adults living with HIV in public health facilities in Geita region, March 2025 (*N* = 423).

Variable	Bivariable poisson (robust SE)			Multivariable poisson (robust SE)		
	cPR	95% CI (cPR)	*p*	aPR	95% CI (aPR)	*p*
Sex						
Male (Ref)	1.0			1.0		
Female	0.91	0.80–1.02	0.132	1.01	0.91–1.13	0.823
Age (in years)						
Adolescents (10 – 19) (Ref)	1.0			1.0		
Adults (20 – 29)	0.82	0.67–1.01	0.067	0.89	0.75–1.07	0.227
Adults (30 – 39)	0.91	0.75–1.0	0.337	0.94	0.81–1.10	0.475
Adults (40 – 49)	0.87	0.71–1.08	0.221	0.82	0.68–0.99	0.046
Adults (50+)	0.81	0.62–1.06	0.125	0.76	0.60–0.97	0.025
Marital status						
Single (Ref)	1.0					
Married	0.94	0.80–1.12	0.508			
Separated/Divorced	1.10	0.88–1.36	0.381			
Occupation						
Others (Ref)	1.0					
Peasants	1.07	0.89–1.28	0.451			
Type of health facility						
Dispensary (Ref)	1.0					
Health center	1.17	0.95–1.43	0.134			
District Hospital	1.06	0.85–1.33	0.593			
Type of clinic						
CTC (Ref)	1.0			1.0		
PMTCT	0.68	0.55–0.86	0.001	0.98	0.79–1.22	0.902
HIV illness stage						
HIV stage three or four (Ref)	1.0					
HIV stage one	1.02	0.86–1.21	0.817			
HIV stage two	1.03	0.86–1.22	0.773			
Duration on ART						
New on treatment (within 6 months) (Ref)	1.0					
Continuing treatment (7 or more months)	1.01	0.82–1.23	0.972			
HIV Viral load suppression status						
HVL not suppressed (Ref)	1.0			1.0		
HVL suppressed	0.89	0.76–0.99	0.040	0.63	0.49–0.79	<0.001
Client stability status						
Unstable (Ref)	1.0			1.0		
Stable	0.83	0.72–0.97	0.016	0.97	0.65–1.44	0.871
History of TB						
Previous TB history (Ref)	1.0			1.0		
Never had TB	0.79	0.69–0.92	0.001	0.81	0.71–0.92	0.001
TB preventive treatment (TPT) status						
Completed TPT (Ref)	1.0			1.0		
On TPT	1.17	0.40–1.12	0.057	1.22	0.94–1.60	0.122
Model of drug collection						
Facility 6 month (Ref)	1.0			1.0		
Facility monthly based	0.79	0.68–0.92	0.002	0.55	0.34–0.84	0.006
Facility 3 month	0.86	0.72–1.04	0.114	0.87	0.74–1.02	0.088
Visit type on last clinical encounter						
Unscheduled (Ref)	1.0			1.0		
Scheduled visit	0.95	0.84–1.09	0.539	0.94	0.83–1.07	0.342
Traced back after LTF	1.30	1.16–1.46	<0.001	1.38	1.06–1.81	0.019
Treatment supporter	0.09	0.02–0.36	<0.001	0.09	0.02–0.34	<0.001

SE (Standard Error), cPR (Crude Prevalence Ratio), 95% CI (95% Confidence Interval), aPR (Adjusted Prevalence Ratio), *p* (*p* value), Ref (Reference category).

Clients on a monthly facility-based drug collection model showed a 45% lower prevalence of being consistently screened for TB at each clinical encounter compared to those on a six-month model (aPR = 0.55; 95% CI = 0.34–0.84; *p* = 0.006). Similarly, individuals on a three-month facility-based drug collection model exhibited a 13% lower prevalence of consistent TB screening at every clinical encounter compared to those on a six-month model, although this association was not statistically significant (aPR = 0.87; 95% CI = 0.74–1.02; *p* = 0.088). Moreover, clients whose treatment supporter collected medication on their behalf at the most recent clinical visit had a 91% lower prevalence of being consistently screened for TB at each encounter compared to those attending in person for either scheduled or unscheduled visits (aPR = 0.09; 95% CI = 0.02–0.34; *p* < 0.001). Individuals traced back from loss to follow-up had a 38% higher prevalence of consistent TB screening compared to those attending a scheduled visit (aPR = 1.38; 95% CI = 1.06–1.81; *p* = 0.019) (Table 5).

Table 6 reports the bivariable and multivariable Modified Poisson regression analyses of factors associated with the correct utilization of the W4SS. Individuals attending PMTCT clinics had 45% lower prevalence of being correctly screened for TB using the W4SS compared to those attending CTCs (aPR = 0.55; 95% CI = 0.39–0.76; *p* < 0.001).

**Table 6:** Modified Poisson regression of factors associated with the correct use of the W4SS tool among adolescents and adults living with HIV in public health facilities in Geita region, March 2025 (*N* = 423).

Variable	Bivariable poisson (robust SE)			Multivariable poisson (robust SE)		
	cPR	95% CI (cPR)	*p*	aPR	95% CI (aPR)	*p*
Client sex						
Male (Ref)	1.0			1.0		
Female	0.89	0.77–1.04	0.146	1.02	0.88–1.18	0.734
Type of health facility						
Dispensary (Ref)	1.0					
Health center	0.90	0.73–1.12	0.345			
District Hospital	0.89	0.70–1.13	0.336			
Type of clinic						
CTC (Ref)	1.0			1.0		
PMTCT	0.55	0.40–0.77	<0.001	0.54	0.39–0.76	<0.001

SE (Standard Error), cPR (Crude Prevalence Ratio), 95% CI (95% Confidence Interval), aPR (Adjusted Prevalence Ratio), *p* (*p* value), Ref (Reference category).

## Discussion

Tuberculosis (TB) screening among people living with HIV (PLHIV) is an essential component of routine clinical services (42, 43). It facilitates early diagnosis and prompt initiation of treatment for TB, thereby improving outcomes and reducing mortality (44). Systematic TB screening increases case detection and helps reduce the overall TB burden among PLHIV. Undiagnosed TB is a leading cause of death in this population and poses a transmission risk to family members and healthcare workers, further contributing to the spread of drug-resistant TB strains (45). The TB screening algorithm requires that all clients identified as presumptive for tuberculosis undergo appropriate laboratory investigations as per national guidelines (14, 18, 46). Those diagnosed with TB should be promptly initiated on treatment, while those who test negative should be assessed for eligibility and offered tuberculosis preventive treatment (TPT) (14). Effective implementation of these steps is critical for interrupting transmission, reducing morbidity and mortality, and achieving programmatic targets in high TB/HIV burden settings (14, 47, 48).

In this evaluation, laboratory investigation results were recorded for 94% of TB presumptive clients, and all individuals diagnosed with TB were successfully initiated on the recommended treatment regimen. These findings indicate a relatively high level of compliance with the recommended screening steps and demonstrate strong fidelity in completion of the TB screening algorithm cascade in this setting. These results are comparable to those from a recent evaluation in Zimbabwe, where 90.5% of PLHIV diagnosed with TB within 12 months received TB treatment (49). However, the performance observed in the current evaluation was notably higher than that reported in Kenya, where 92% of symptomatic PLHIV did not have laboratory results recorded during their encounters (43). Our findings were also higher than what was reported in another evaluation conducted in Tanzania where 31.59% of clients who screened positive for TB symptoms had no documented follow-up investigations (12).

The relatively high fidelity observed in our evaluation may reflect improvements in health system performance, especially in the diagnostic services and follow-ups. Ongoing quality improvement initiatives, supportive supervision, and better integration between TB and HIV services at the facility level may have supported these improvements. Overall, while challenges and suboptimal performance were reported in the initial stages of symptom screening, the completion of the diagnostic and treatment steps in the cascade appears to be well-implemented in the study area. Strengthening fidelity at earlier stages of the algorithm, such as the consistent and correct use of the W4SS, may further improve TB case detection, treatment outcomes and reduction of TB morbidity in this population.

However, fidelity to the screening algorithm was suboptimal at the initial stages of symptom screening. Our evaluation showed that TB screening was documented in 82.8% of clinical encounters over a one-year period. With only 70.7% of individuals consistently screened during all their clinic visits, and 75.4% screened during their most recent visit. These results suggest that a significant proportion of HIV-positive individuals were not consistently screened for TB as required, reflecting a suboptimal adherence to the screening guidelines (14, 50). This underperformance falls short of both WHO recommendations and Tanzania’s national guidelines, which mandate TB screening at every clinic visit for adolescents and adults living with HIV (14, 50).

Furthermore, the WHO recommends that all adolescents and adults living with HIV should be screened for TB using either the W4SS tool or chest X-ray (CXR) as provided in the screening algorithm (14). According to both WHO and national guidelines, when using the W4SS tool, healthcare providers are required to ask four specific symptom-based questions to every adolescent and adult client during each clinic visit (14, 18, 46). In this evaluation, the W4SS was the commonly used tool in over 98% of the screening sessions. However, healthcare providers correctly administered all four screening questions in only 62% of the sessions observed. This indicates that 38% of clients were not adequately screened for TB according to the established guidelines (14, 18, 46). Malpractices in the screening practices were common, where healthcare providers frequently asked only one question – typically about coughing – or used vague general inquiries such as “Do you have any problem?” In some cases, no TB screening questions were asked at all. These in accurate application of the W4SS tool reflect further suboptimal fidelity to the screening algorithm, at the initial symptom screening stages.

Low uptake of the TB screening algorithm has also been reported in other high TBHIV settings in the sub-Saharan African countries (45, 49). For example, in Ghana, a substantial proportion (36.81%) of HIV-positive clients had no documentation of TB screening in their medical records (45). A study in Zimbabwe found that only 48.7% of adult PLHIV reported having been screened for TB symptoms at their last HIV care visit (49). In Kenya, TB screening was recorded in 87% of the clinical encounters among PLHIV in the study (43). These figures reflect broader challenges in the consistent application of TB screening protocols in routine HIV care in the high TBHIV countries in SSA.

On the other side, the findings of our study were lower than what was reported in Ethiopia where TB screening was reported among 90.5% of the HIV clients (30). Our findings were also lower than what was reported in another study in Tanzania that found screening records documented in 95.7% of the clients in the electronic medical records (12). The lower coverage observed in our study may be partly attributed to differences in data sources. While the previous Tanzanian study used electronic databases (12), our evaluation relied on paper-based treatment records, the source documents at the health facilities.

The limited utilization of the CXR screening test observed in our evaluation, may be attributed to the cost associated with accessing the service, as clients are required to pay for the film. Given the epidemiology of TB with the disease being more prevalent among the poor (51, 52), it is likely that many clients would not afford CXR services when needed. Similar observations were also reported in another study conducted in East Africa countries, including Tanzania, where Mnyambwa et al. (48) reported that the requirement of clients to pay for the X-ray services was one of the barriers for consistent TB screening.

This evaluation examined the factors associated with TB screening at every clinical encounter and the correct application of the W4SS tool. The findings revealed that increasing age was significantly associated with lower prevalence of being consistently screened for TB at every clinic visit among clients. Specifically, adults older than 40 years were less likely to receive consistent TB screening during each encounter compared to adolescents. While epidemiological evidence suggests that older adults are at higher risk of developing TB due to age-related immunosuppression and comorbidities (53), the current study observed a contrary trend, where adolescents were more likely to be consistently screened. This could be explained by the presence of targeted HIV interventions focusing on adolescents and youth as a vulnerable group. These interventions may inadvertently result in healthcare providers giving greater attention to adolescent clients, leading to higher screening fidelity in this subgroup. These findings are consistent with another study conducted in Kenya, which similarly reported older clients had a higher risk of being inadequately screened (43). These patterns indicate need for healthcare systems to ensure equitable service delivery across all age groups, especially older adults who remain highly vulnerable to TB infection and progression.

In this evaluation, clients attending Prevention of Mother-to-Child Transmission (PMTCT) clinics were found to be less likely to be consistently screened for TB at every clinical encounter compared to those attending Care and Treatment Clinics (CTCs). A similar trend was observed in the correct use of the W4SS tool, where healthcare providers in PMTCT settings had a significantly lower prevalence of administering the screening questions correctly compared to their counterparts in CTCs. This finding may be attributed to higher workloads and limited staffing in PMTCT units. This aligns with findings from (48), who reported that fewer than one-third of health facilities in East Africa had successfully integrated TB screening activities into their reproductive and child health (RCH) departments.

However, the results of this evaluation contrast with findings from Owiti et al. (43) in Kenya, where PLHIV seen in PMTCT clinics were less likely to experience undesirable screening outcomes compared to those in general HIV care clinics (CTCs). This discrepancy may reflect differences in health system structures, human and financial resource allocation between Tanzania and Kenya. These findings suggest a need for more efforts to strengthen TB screening within PMTCT services, ensuring that pregnant and breastfeeding women living with HIV receive the recommended quality screening services.

The status of HIV viral load suppression was found to significantly influence the fidelity of TB screening algorithm implementation in this evaluation. Clients with suppressed HIV viral loads were less likely to be screened for TB at every clinical encounter compared to those with unsuppressed viral loads. This trend may be attributed to healthcare providers’ perceptions that clients with stable HIV conditions – including those with viral suppression – are at lower risk of developing active TB.

Similar findings were observed by Maokola et al. (12) where clients with stable clinical presentations were less likely to be screened for TB compared to those with advanced clinical stages, such as those with WHO stage IV. Similar findings were also reported in Ghana, where clients with high hemoglobin and those with a high CD4 + T cell count were negatively associated with the performance of TB screening (45). These patterns suggest that clinical stability may unintentionally lead to relaxed adherence to the screening protocols, despite guidelines recommending universal TB screening for all PLHIV regardless of clinical or immunological status. This emphasizes the importance of continued efforts to reinforce guideline adherence and raise provider awareness about the continued risk of TB among virally suppressed clients.

The prevalence of consistence TB screening at every clinical encounter were significantly lower among individuals without a prior history of tuberculosis compared to those with such a history. Similar trends have been reported in studies from Ghana and Ethiopia, where clients with chronic cough or a known history of TB had greater odds of being screened (30, 45). This pattern may be explained by healthcare providers’ higher attention or heightened vigilance toward clients with previous TB episodes, given the evidence that individuals with a history of TB are at increased risk of developing another episode. People successfully completing treatment for tuberculosis remain at elevated risk for recurrent disease, either from relapse or reinfection due to lung damage or immune compromise, which increase the risk of relapse or reinfection (54). It is likely that healthcare providers in the study setting are aware of this epidemiological risk and, as a result, prioritize consistent and thorough TB screening for clients with a known TB history. Such practices reflect appropriate clinical judgment and may contribute to timely identification of recurrent TB, which is critical in high-burden settings (30).

In this evaluation, a statistically significant association was found between the drug collection model and consistent TB screening at every clinical encounter. Clients enrolled in monthly or three-month facility-based drug collection models had lower prevalence of being consistently screened for TB compared to those on the six-month facility-based model. These findings contrast with results from a study conducted in Ghana, where newly diagnosed HIV infections were associated with increased odds of TB screening (45). The divergence observed in the current study may be explained by healthcare providers’ screening practices. Specifically, providers may demonstrate greater attentiveness to clients on longer refill intervals, such as those on the six-month model, because these individuals return to the facility less frequently. As a result, each visit is seen as a critical opportunity to conduct comprehensive clinical assessments, including TB screening.

Conversely, clients on monthly or three-month refill schedules may be perceived as being under more regular clinical observation, potentially leading providers to deprioritize TB screening during each visit. Additionally, these clients may be more likely to be currently receiving or have recently completed TPT, which could reinforce the perception among providers that they are at lower risk for TB, thereby reducing the perceived necessity for routine screening. These findings emphasize on the continued need to reinforce screening guidelines across all models of care, ensuring that routine TB screening is consistently implemented regardless of a client’s refill schedule or perceived clinical stability as required by the guidelines (14, 46).

Individuals traced back from loss to treatment follow-up (LTF) were found to be more consistently screened for TB compared to those who had remained in regular care. This finding may reflect the heightened clinical attention and comprehensive reassessment typically provided to clients who return to care after treatment interruption (55, 56). Clients who return from LTF are more at risk of poor treatment outcomes including low viral suppression and higher chance of developing active TB (55–57). Thus, when clients are re-engaged, healthcare providers may often conduct a full clinical evaluation, including TB symptom screening, as part of the routine re-initiation process (57). Moreover, these clients are often prioritized for intensified monitoring and follow-up to prevent further disengagement, such initiative may further enhance adherence to standard screening protocols. This observation calls on the importance of maintaining similar rigor in TB screening for all clients, not only those traced from LTF.

Screening for TB at every clinical visit was found to be less consistent among clients whose medication was collected by a treatment supporter at some point during the previous 12 months. This finding is expected, as TB screening using either the W4SS or the CXR requires the physical presence of the client for symptom assessment and clinical interaction or taking the chest X-ray film (14). When a treatment supporter collects medication on behalf of the client, the opportunity for routine TB screening is missed. This highlights the importance of ensuring that alternative follow-up mechanisms are in place to monitor TB symptoms in clients who are unable to attend clinic visits in person.

## Conclusion

While completion of the algorithm cascade was well adhered to – with laboratory investigations conducted, all TB patients initiated on treatment, and eligible clients started on TPT – fidelity to the screening algorithm was suboptimal at the initial stage of symptom screening. This was evidenced by inconsistent screening and deviations from the use of the recommended W4SS tool. Factors significantly associated with inconsistent screening included older age, suppressed HIV viral load, attendance at PMTCT clinics, history of previous TB, being traced from a loss to follow up, and being on a monthly refill model. To improve adherence to the screening protocol, especially at the symptom screening stage, focused training and targeted monitoring should be provided emphasizing accurate and mandatory use of the W4SS tool at every encounter. Role-playing should be used to avoid vague screening questions.

### Potential study limitations and mitigation

Participants on the observation component might have altered their behavior – regarding their adherence to the TB screening algorithm – if they became aware that they were being observed, the *Hawthorne effect* (58). Such behavior could lead to an overestimation of fidelity during the observation sessions. To mitigate this, covert observation was employed; healthcare providers were not explicitly informed that their use of the TB screening algorithm was the focus of the observation. This approach was intended to minimize changes in natural behavior and improve the accuracy of the observed data.

Although the study included regression analyses with multiple predictors, the sample size was originally calculated to estimate proportions rather than to power multivariable modeling. Consequently, some associations may have been underpowered, and the results should therefore be interpreted with caution. To mitigate potential overfitting, we limited the number of variables included in the multivariable models to those with clinical relevance with non-collinearity, and we also assessed model stability prior to final model selection.

Moreover, the study partly relied on secondary data sourced from routine health facility records, including TB screening forms and patient treatment records (CTC2 files). The use of secondary data may pose risks such as inaccurate documentation, missing data, or incomplete records, which could affect the reliability of the findings. To address this limitation, we added a 10% percent non response rate to the sample size and we excluded all missing values on analysis.

## Data Availability

The raw data supporting the conclusions of this article will be made available by the authors, without undue reservation.

## Author’s note

LS was a postgraduate student pursuing MSc. Health Monitoring and Evaluation under the supervision of LM and MA. LM was a senior lecturer and director of quality assurance at Mzumbe University, MA was a professor and head of the department of Health Systems Management at Mzumbe university.
