# Supplementary material for: Provider fidelity in tuberculosis screening practices among adolescents and adults living with HIV in public health facilities in Tanzania: a cross-sectional evaluation
Source: Front Public Health. 2025 Nov 19;13:1688829. doi: 10.3389/fpubh.2025.1688829 (PMC12672541; doi:10.3389/fpubh.2025.1688829)
Supplement: Supplementary file 1 [file Table_1.docx]

**Supplementary Table 1:**

**The extent of TB screening during clinical encounters over a 12 months period among adolescents and adults living with HIV in public health facilities in Geita region (N = 423).**

| **Variable** | **Categories** | **Total** | | **Adolescent** | | **Adult** | | **P** |
| --- | --- | --- | --- | --- | --- | --- | --- | --- |
|  |  | **N** | **%** | **N** | **%** | **N** | **%** |  |
| Individual screened in all clinical encounters in the past 12 months | No | 124 | 29.3 | 7 | 19.4 | 117 | 30.2 | 0.174 |
|  | Yes | 299 | 70.7 | 29 | 80.6 | 270 | 69.8 |  |
| Individual screened in the recent clinical encounter | No | 104 | 24.6 | 7 | 19.4 | 97 | 25.1 | 0.454 |
|  | Yes | 319 | 75.4 | 29 | 80.6 | 290 | 74.9 |  |
| Screening test used in recent clinical encounter | Chest X-ray | 6 | 1.9 | 0 | 0 | 6 | 2.1 | 0.433** |
|  | W4SS | 313 | 98.1 | 29 | 100 | 284 | 97.9 |  |
|  | Not Screened* | 104 | - | 7 | - | 97 | - |  |
| W4SS screening form is available in the client CTC2 file | No | 20 | 4.7 | 2 | 5.6 | 18 | 4.7 | 0.807 |
|  | Yes | 403 | 95.3 | 34 | 94.4 | 369 | 95.3 |  |

**Key:** N (Number), % (Percentage), P (p value), * (not included in calculation of percentages), ** (Fisher’s exact test – one cell has zero observation)
