# Supplementary material for: Provider fidelity in tuberculosis screening practices among adolescents and adults living with HIV in public health facilities in Tanzania: a cross-sectional evaluation
Source: Front Public Health. 2025 Nov 19;13:1688829. doi: 10.3389/fpubh.2025.1688829 (PMC12672541; doi:10.3389/fpubh.2025.1688829)
Supplement: Supplementary file 2 [file Table_2.docx]

**Supplementary Table 2:**

**The extent to which healthcare providers complete the recommended steps of the screening algorithm for detecting TB among adolescents and adults living with HIV in public health facilities in Geita, March 2025 (N = 423)**

| **Variable** | **Categories** | **Total** | | **Adolescent** | | **Adult** | | **P** |
| --- | --- | --- | --- | --- | --- | --- | --- | --- |
|  |  | **N** | **%** | **N** | **%** | **N** | **%** |  |
| Presumptive TB cases have the laboratory investigation results documented | No | 6 | 6.0 | 0 | 0 | 6 | 6.6 | 0.427** |
|  | Yes | 94 | 94.0 | 9 | 100.0 | 85 | 93.4 |  |
|  | Never screened presumptive* | 323 | - | 27 | - | 296 | - |  |
| Individual diagnosed with TB started the recommended ant TB treatment | No | 0 | 0 | 0 | 0 | 0 | 0 | N/A |
|  | Yes | 43 | 100.0 | 4 | 100.0 | 39 | 100.0 |  |
|  | Never diagnosed with TB* | 380 | - | 36 | - | 387 | - |  |
| Individuals with negative TB who were eligible for TPT initiated the recommended TPT on their recent clinical encounter | No | 7 | 19.4 | 0 | 0 | 7 | 20.0 | 0.618** |
|  | Yes | 29 | 80.6 | 1 | 100.0 | 28 | 80.0 |  |
|  | Not eligible for TPT* | 387 | - | 35 | - | 352 | - |  |

**Key:** N (Number), % (Percentage), P (p value), * (not included in calculation of percentages), ** (Fisher’s exact test – one cell has zero observation), N/A (Not Applicable – The comparison group has zero observation).
